# Characterization of Household and Community Shedding and Transmission of Oral Polio Vaccine in Mexican Communities With Varying Vaccination Coverage

**DOI:** 10.1093/cid/ciy650

**Published:** 2018-10-30

**Authors:** Jonathan Altamirano, Natasha Purington, Rasika Behl, Clea Sarnquist, Marisa Holubar, Lourdes García-García, Leticia Ferreyra-Reyes, Rogelio Montero-Campos, Luis Pablo Cruz-Hervert, Shanda Boyle, John Modlin, Christopher van Hoorebeke, Sean Leary, ChunHong Huang, Marvin Sommer, Elizabeth Ferreira-Guerrero, Guadalupe Delgado-Sanchez, Sergio Canizales-Quintero, José Luis Díaz Ortega, Manisha Desai, Yvonne A Maldonado

**Affiliations:** 1 Stanford University School of Medicine, California; 2 Instituto Nacional de Salud Pùblica, Cuernavaca, Mexico; 3 Bill & Melinda Gates Foundation, Seattle, Washington; 4 Universidad Nacional Autónoma de México (UNAM), Mexico City, Mexico

**Keywords:** OPV, shedding, oral poliovirus vaccine, poliovirus transmission, longitudinal virus surveillance, Mexico

## Abstract

**Background:**

The World Health Assembly 2012 Polio Eradication and Endgame Strategic Plan calls for the eventual cessation of all oral polio vaccines (OPVs), to be replaced with inactivated polio vaccine (IPV); however, IPV induces less robust mucosal immunity than OPV. This study characterized household and community OPV shedding and transmission after OPV vaccination within primarily IPV-vaccinated communities.

**Methods:**

Households in 3 IPV-vaccinated Mexican communities were randomized to receive 3 levels of OPV vaccination coverage (70%, 30%, or 10%). Ten stool samples were collected from all household members over 71 days. Analysis compared vaccinated subjects, household contacts of vaccinated subjects, and subjects in unvaccinated households. Logistic and Cox regression models were fitted to characterize transmission of OPV by coverage and household vaccination status.

**Results:**

Among 148 vaccinated children, 380 household contacts, and 1124 unvaccinated community contacts, 78%, 18%, and 7%, respectively, shed OPV. Community and household contacts showed no differences in transmission (odds ratio [OR], 0.67; 95% confidence interval [CI], .37–1.20), in shedding trajectory (OR, 0.61; 95% CI, .35–1.07), or in time to shedding (hazard ratio, 0.68; 95% CI, .39–1.19). Transmission began as quickly as 1 day after vaccination and persisted as long as 71 days after vaccination. Transmission within unvaccinated households differed significantly across vaccination coverage communities, with the 70% community experiencing the most transmissions (15%), and the 10% community experiencing the least (4%). These trends persisted over time and in the time to first shedding analyses.

**Conclusions:**

Transmission did not differ between household contacts of vaccinees and unvaccinated households. Understanding poliovirus transmission dynamics is important for postcertification control.

Live, attenuated oral polio vaccine (OPV) has been used for several decades as the primary polio vaccine in developing countries. OPV recipients shed vaccine virus in their stool, which confers herd immunity, considered to be among the greatest attributes of the vaccine [1]. Previous studies have found that OPV-vaccinated children typically shed the virus for 4–8 weeks, with a few extreme cases in which an immunocompromised individual has shed the virus for months or even years [2, 3]. Exposure to poliovirus from vaccinated children can lead to household contact shedding, which can then lead to more widespread community circulation of poliovirus [4, 5]. A study conducted in Bihar, India, found statistically significantly higher shedding in index households than in neighborhood households (38.9% vs 7.9%, respectively), and a previous study conducted by our research group found that the OPV continued to circulate within households for up to 7 months after an OPV vaccination campaign in rural Mexico [6].

As we anticipate global eradication of polio, polio vaccine–related shedding is no longer desirable [7]. Furthermore, OPV virus is genetically unstable, evolving during circulation within vaccinated communities, and is the underlying cause of vaccine-associated paralytic poliomyelitis in OPV recipients and their close contacts [8, 9]. More concerning is that long-term replication and mutation of OPV in the gastrointestinal tract can lead to genetically divergent vaccine-derived polioviruses (VDPVs). This is of particular concern in undervaccinated communities and, therefore, low circulating immunity and increased susceptibility to infection and resulting disease. The average incidence of VDPVs between 2005 and 2013 was approximately 76 cases annually [8], and 6 countries were affected by VDPV outbreaks in 2015 [9]. By contrast, as of December 2017, only 22 cases of wild poliovirus (WPV) have been confirmed, a decline from 37 cases in 2016 [10]. Of concern is circulation of OPV serotype 2, because our group has shown this serotype can be detected in sewage as long as 7 months after vaccination, indicating prolonged circulation [11].

The World Health Assembly declared in 2012 that the eradication of polio constitutes “a programmatic emergency for global public health” and released the Polio Eradication and Endgame Strategic Plan 2013–2018. That plan aims to simultaneously achieve the eradication of WPV and the elimination of VDPV [12]. VDPVs have been shown to cause paralysis similar to that caused by WPV in outbreaks, as shown by the 84 paralytic cases of circulating VDPV serotype 2 isolated in 2017 after outbreaks in the Democratic Republic of the Congo and Syria [10]. To achieve the goals of WPV eradication and elimination of VDPVs, a strategy is outlined to gradually withdraw all use of OPVs after the eradication of WPV so that the VDPVs remaining in circulation do not cause outbreaks of polio in a new generation of unvaccinated children. This plan recommends replacing OPV with inactivated polio vaccine (IPV), overlapping each vaccine’s use in most countries, with every country introducing ≥1 dose of IPV by 2016 [13].

However, factors affecting OPV transmission within and between households of vaccinated children are still not well understood. Therefore, the current study aims to characterize the incidence and duration of OPV shedding and transmission of vaccinated children to their household and community contacts. Specifically, overall shedding and transmission, shedding and transmission over time, and time to first shedding are explored. Mexico provides a natural environment to study polio vaccine transmission in a dual IPV-OPV environment, because it introduced IPV into its routine childhood vaccination schedule in 2007 but continues to give OPV twice a year during National Health Weeks (NHWs) [6].

## METHODS

### Study Design

The study began in December 2015 with a census of all households in 3 Mexican localities in Orizaba, Veracruz (Capoluca, Campo Grande, and Tuxpanguillo). A total of 466 households, approximately 150 from each community, were enrolled to participate during and after the February 2015 NHW. All households had a healthy child ≤5 years old with up-to-date IPV vaccinations, because only children <5 years old are eligible for OPV vaccination in Mexico. Households were randomized in parallel within each community to either receive OPV or remain unvaccinated. If a household contained multiple children ≤5 years old, only 1 was randomly selected to receive OPV during the February 2015 NHW. No other OPV was introduced in these communities until the May 2015 NHW.

Each locality received different OPV coverage levels—70% of study households in Capoluca, 30% in Campo Grande, and 10% in Tuxpanguillo—to assess for differences associated with different OPV vaccination coverage. Ten stool samples were collected from each study participant, 1 sample at baseline before the first NHW and 9 samples collected serially over 71 days (days 1, 4, 7, 10, 14, 21, 28, 51, and 71). These samples were analyzed by means of reverse-transcription polymerase chain reaction (PCR) for OPV serotypes to assess OPV shedding and transmission in these communities. Detailed methods involved in the study design have been published elsewhere [14].

Viral RNA was extracted from frozen stool samples after thawing, using the MagNA Lyser instrument (Roche) and the KingFisher Duo Prime system (Fisher Scientific), using the bacteriophage MS2 as an internal control for extraction efficiency. Viral RNA was then analyzed using quantitative reverse-transcription PCR to detect and quantify any Sabin OPV present in the samples. The probes and primers were adopted and adapted from Kilpatrick et al [15] and the Centers for Disease Control and Prevention protocol for polio quantitative PCR. Samples were analyzed in triplicate and a sample was considered positive if 2 of 3 reactions had a cycle threshold <37. Positive samples were analyzed again, to minimize false-positives, and if results were again positive, the RNA was Sanger sequenced for confirmation.

Written informed consent was obtained from all adult participants, parents or guardians of minors consented for participating minors (children ≤18 years of age), and assent forms were obtained from children 7–18 years of age. The study protocol was reviewed and approved by (1) Stanford University’s Institutional Review Board (protocol 31546), (2) the Comité de Etica, Bioseguridad e Investigación of the Instituto Nacional de Salud Pública (CI 1260; No. 1581), and (3) the Instituto Veracruzano Para la Formación e Investigación en Salud (SESVER/IVEFIS//SIS/DIB/0109/02014; classification 15S). This trial is registered at ClinicalTrials.gov (NCT02376374).

### Outcome Measures

There were 3 primary outcomes that corresponded to 3 objectives. The first was an indicator for detection of OPV in any stool sample of a vaccinated children or household contact at any time during the study period, to assess overall transmission to household contacts. The second was an indicator for detection of poliovirus in stool samples at a given time point to identify differences in intrahousehold OPV transmission over time. The third was the time to the first positive stool sample. All 3 outcome measures were defined for overall OPV shedding and shedding by serotype and focused on postbaseline samples.

### Key Variables of Interest

There were 3 variables of interest involved in addressing our objectives. Specific community membership (ie, living in the communities with 705, 30%, or 10% vaccination), time to virus transmission from study enrollment, and household vaccination status (vaccinated or unvaccinated household). Subjects in vaccinated households and unvaccinated households are referred to as “household contacts” and “community contacts,” respectively. Preliminary analyses were conducted separating the vaccinated children into their 3 coverage areas, but shedding patterns among these groups were comparable. Therefore, owing to the homogeneity of shedding among vaccinated children, vaccinees were treated as a single group.

### Statistical Analysis

Descriptive statistics such as means, medians, standard deviations (SDs), and interquartile ranges were generated to evaluate distributions of key variables. Duration of shedding was compared between groups using Wilcoxon-Mann-Whitney tests. Two-tailed Fisher exact tests were used to compare shedding rates between children ≤5 years old with differing prior exposure to OPV and IPV. Children with any prior OPV exposure were compared with children without prior OPV exposure and children with 3 doses of IPV were compared with those with 4 doses. (Children with <3 doses were not included owing to smalls sample sizes.)

To examine overall transmission, logistic regression models were fit to transmission at any point in the study as a function of vaccination coverage, household vaccination status, and their interaction. The significance of the interaction term determined whether transmission differed in each coverage area by household vaccination status.

To assess these same associations over time, longitudinal logistic models were fit to shedding/transmission at a certain time as a function of coverage area/vaccination status (CV) group (eg, Vaccinees, 10% Household Contacts, etc), time in days, quadratic time, and the interaction between CV group and each time variable. Quadratic time, or time squared, is a term added to regression analyses when an outcome is quadratic over time, such as shedding of OPV, which rises, peaks, and then declines over time. The significance of the interaction terms determined whether the different CV groups showed significantly different transmission by vaccine coverage. Global *P* values were presented, which tested the interaction parameters jointly, determining whether shedding/transmission had different trajectories across CV group. Owing to the small number of events after 28 days, a sensitivity analysis was performed on the longitudinal models restricting to the first 28 days of the study.

To determine whether time to first shedding/transmission differed across the various groups, Cox proportional hazards models were fit to time to first shedding/transmission as a function of coverage, household vaccination status, and their interaction. Owing to subjects clustering within families and repeated measures on subjects over time, the marginal model approach using an exchangeable correlation structure was used to address our research questions. This approach is generally used in the analysis of correlated data [16]. Failing to account for possible correlation can lead to underestimation of the variance, which can result in artificially low *P* values [17]. Therefore, to account for household clusters, a household cluster effect was included in the overall shedding models as well as the survival models. To account for household clusters and repeated measures on subjects over time, a subject nested in household cluster effect was included in the longitudinal logistic models.

All analyses were conducted for overall OPV and by serotype, and also adjusted for continuous age, whether or not the house had a running toilet, and household density (number of persons living in the house). Odds ratios (ORs) or hazard ratios (HRs) and 95% confidence intervals (CIs) for household contacts versus vaccinated children and each pairwise comparison within household contacts were reported. Differences considered statistically significant at *P* < .05. All analyses were conducted using SAS statistical software, version 9.4 (SAS Institute).

## RESULTS

### Baseline Characteristics

The 1652 subjects included in the analyses included 148 vaccinated children, 380 unvaccinated household contacts in vaccinated households (household contacts) and 1124 unvaccinated subjects in unvaccinated households (community contacts) who provided ≥1 postbaseline fecal sample during the study period. The distribution of vaccinated households in each of the 3 localities was consistent with that outlined in the study protocol, with 91 (61%) households vaccinated in the 70% coverage area, 41 (28%) in the 30% area, and 17 (11%) in the 10% area (Table 1).

**Table 1.** Baseline Characteristics of 3 Indigenous Mexican Study Villages

Baseline Characteristic	Coverage Area/Vaccination Status Group						
	Vaccinees	70% Coverage		30% Coverage		10% Coverage	
		Household Contact	Unvaccinated Households	Household Contact	Unvaccinated Households	Household Contact	Unvaccinated Households
Subjects, No.	148	216	164	115	375	49	585
Households, No.	…	91	50	41	94	17	145
Age, mean (SD), y	2.7 (1.2)						
Subjects by age group, No. (%)							
Adults (≥18 y old)	…	29.8 (9.6)	32.1 (13.9)	32.7 (11.0)	32.7 (11.0)	28.6 (5.6)	37.0 (15.5)
Children 6–17 y old	…	11.0 (3.3)	9.4 (3.0)	11.5 (3.6)	10.5 (3.6)	9.0 (2.7)	10.5 (3.6)
Children ≤5 y old	…	2.5 (1.6)	2.7 (1.3)	1.4 (0.8)	2.5 (1.3)	1.7 (2.4)	2.8 (1.3)
Female sex, No. (%)	72 (49)	146 (68)	102 (62)	69 (60)	214 (57)	31 (63)	346 (59)
Type of family member, No.							
Women	…	99	59	43	120	20	201
Men	…	33	14	29	63	9	91
Female children >5 y old	…	37	14	19	33	6	45
Male children >5 y old	…	34	18	16	37	7	59
Other children ≤5 y other than vaccinee	…	13	59	8	122	7	189
Household density, mean (SD), No. of persons in house^b^	…	4.3 (1.5)	4.3 (1.2)	4.6 (1.7)	4.7 (1.6)	4.4 (1.2)	4.9 (1.7)
Household features, No. (%)							
Running water^b^	…	72 (79)	41 (82)	39 (95)	80 (85)	16 (94)	129 (89)
Toilets with running water^b^	…	71 (78)	33 (66)	37 (90)	67 (71)	16 (94)	129 (89)
Cement floors^b^	…	81 (89)	42 (84)	34 (83)	83 (88)	12 (71)	129 (89)
Electricity^b^	…	87 (96)	49 (98)	40 (98)	93 (99)	16 (94)	142 (98)
Parents speaking indigenous language, No. (%)^b^	…	23 (25)	16 (32)	2 (5)	3 (3)	9 (53)	80 (55)
Social development wealth index^c^	…	3		2		2	
Prior OPV exposure, No. (%)	116 (78)	8 (62)	40 (68)	2 (25)	81 (66)	1 (14)	133 (70)
IPV doses, No. (%)							
0 doses	…	2 (15)	2 (3)	…	3 (2)	1 (14)	5 (3)
1 dose	…	…	…	…	2 (2)	1 (14)	1 (1)
2 doses	1 (1)	…	1 (2)	2 (25)	5 (4)	…	3 (2)
3 doses	42 (28)	2 (15)	11 (19)	1 (13)	44 (36)	…	47 (25)
4 doses	105 (71)	8 (62)	43 (73)	3 (38)	60 (49)	1 (14)	121 (64)
Samples collected, No.	1359	1927	1479	938	3243	478	5629
Samples collected per person, mean (SD)	9.1 (1.5)	8.8 (2.3)	8.8 (1.9)	8.1 (2.3)	8.5 (2.1)	9.8 (0.5)	9.6 (1.1)

If the cell is blank, there were no participants within that category.

Abbreviations: IPV, inactivated polio vaccine; OPV, oral polio vaccine; SD, standard deviation.

^a^All percentages are based have the total as the denominator unless otherwise noted.

^b^Calculated on the household level.

^c^For the community overall.

The age distributions across the household contacts were comparable. The average age in unvaccinated households varied between communities, from an average of 32 years for adults in the 70% to 37 years for adults in the 10% community. Sex ratios and household density across groups were also comparable. Households in the 70% and 30% communities had lower prevalences of toilets with running water (74% and 77%, respectively) than those in the 10% communities (90%; Table 1). Asset ownership and wealth quintiles could not be analyzed using the Demographic and Health Surveys, because data have not been collected in Mexico since 1987 [18]. Instead, we used a similar 5-point (1, lowest; 5, highest) social development wealth index created by the Mexican National Council for Evaluation of Social Development Policy [19]. This index found that the 30% and 10% communities have a low score of 2, and the 70% community has a middle score of 3. A total of 13332 samples were provided, with the average number of samples per subject ranging from 8.1 (household contacts in 30% coverage area) to 9.8 (household contacts in 10% coverage area).

### Overall Household Shedding and Transmission

**Figure 1.**
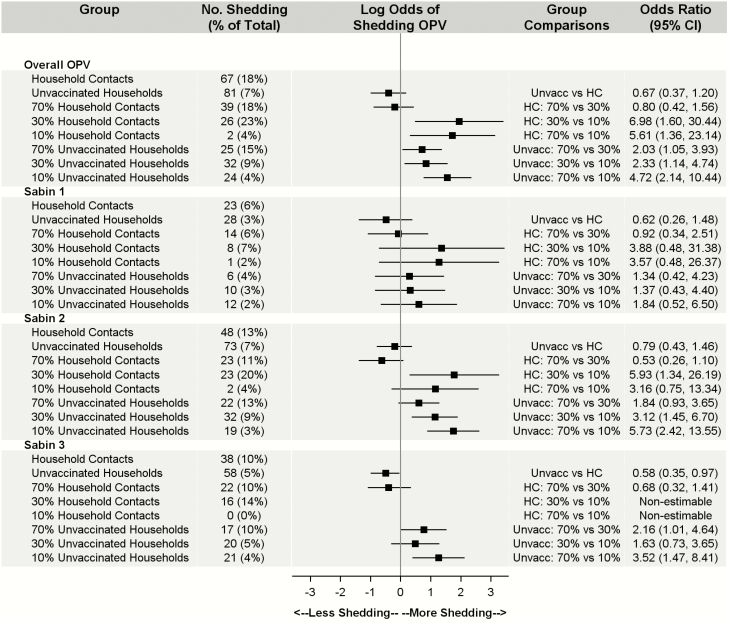
Count and percentage of household contacts (HCs) versus unvaccinated (Unvacc) households positive for oral polio vaccine (OPV) by coverage and serotype. Note that percentages are calculated as the number of subjects with ≥1 probable positive OPV sample divided by the total number of subjects for each group and strain (N = 1652). Models controlled for age, household density, and running toilets with a household cluster effect. Owing to zero household transmission of Sabin 3 in the 10% area, pairwise comparisons between household contacts in the 10% coverage community and the 30% and 70% coverage communities were nonestimable. Abbreviation: CI, confidence interval.

Of the 528 total subjects within vaccinated households, 183 (35%) shed OPV at any point—78% of vaccinated subjects versus 18% of household contacts. Among the contacts, subjects in the 30% community had the highest rate of transmission (23%), although the difference compared with the 70% community was not statistically significant (overall OR, 0.80; 95% CI, .42–1.56) (Figure 1). As expected, household contacts had a significantly lower probability of shedding any OPV than vaccinated children (OR, 0.04; 95% CI, .02–.09). This association persisted across serotypes (OR for Sabin serotype 1, 0.03 [95% CI, .01–.08]; Sabin 2, 0.06 [.03– .13]; Sabin 3, 0.17 [.09–.33] (Supplementary Figure 1). Compared with the 10% area, transmission within household contacts was >5 times higher in the 70% area (overall OR, 5.61; 95% CI, 1.36–23.14) and almost 7 times higher in the 30% area (6.98; 1.60–30.44). Apart from the 30% versus the 10% area for serotype 2 (OR, 5.93; 95% CI, 1.34–26.19), these differences were not observed across serotype. Within serotypes, shedding and transmission were most prevalent in Sabin 2. Age, presence of a running toilet within the household, and household density were not significant predictors of shedding OPV.

Both overall and across serotypes, women in OPV-vaccinated households were the household contacts most likely to shed transmitted OPV. Specifically, 21% of women (34 of 162) shed OPV, accounting for 40%–50% of all transmissions across serotypes (Table 2). Other children in the household (other than the vaccinated child) were the next most susceptible group, with shedding in 15% of children (22 of 147), accounting for 20%–30% of transmissions. Finally, men in the household were the least susceptible, with shedding in 15% (11 of 71), accounting for 16%–30% of household transmissions.

**Table 2.** Shedding and Transmission Summary Statistics

Summary Statistic	Overall	Sabin 1	Sabin 2	Sabin 3
Peak day of shedding/transmission after vaccination				
Vaccinated children	4	4	4	4
Household contacts				
70% Coverage area	7	28	7	21
30% Coverage area	4	4	4	4
10% Coverage area	…	…	…	…
Unvaccinated				
70% Coverage area	28	21	28	28
30% Coverage area	7	4	7	4
10% Coverage area	7	7	7	7
Duration of shedding for vaccinated children, mean (SD), d^a^				
Overall	9.3 (7.1)	7.1 (5.3)	7.3 (5.4)	11.02 (7.4)
No OVP exposure	14.3 (8.4)^b^	10.3 (7.5)^c^	10.3 (6.0)^d^	15.3 (7.7)^b^
≥1 OPV dose	7.6 (5.7)^b^	5.7 (3.1)^c^	5.7 (4.3)^d^	7.8 (5.3)^b^
3 IPV doses	13.6 (8.3)^d^	9.9 (6.8)^b^	9.9 (6.5)^b^	14.0 (8.0)^c^
4 IPV doses	6.9 (4.8)^d^	5.1 (2.6)^b^	5.6 (4.0)^b^	7.9 (4.8)^c^
Time to first shedding/transmission, median (range), d				
Vaccinated children	4 (1–21)	3 (1–28)	4 (1–21)	4 (1–21)
Household contacts				
70% Coverage area	14 (1–75)	17.5 (4–75)	10 (1–74)	17 (4–75)
30% Coverage area	7 (2–73)	4 (2–7)	7 (2–73)	8.5 (3–51)
10% Coverage area	30.5 (10–51)	10 (10–10)	30.5 (10–51)	…
Unvaccinated				
70% Coverage area	21 (1–68)	15 (1–21)	21 (1–68)	27 (1–68)
30% Coverage area	7 (1–52)	5.5 (3–14)	7 (1–52)	10.5 (3–52)
10% Coverage area	14 (1–73)	7 (1–27)	7 (1–73)	7
Transmission in vaccinated household, No. (%)				
Total transmissions	67	23	48	38
Women	34 (51)	11 (48)	22 (46)	16 (42)
Men	11 (16)	7 (30)	10 (21)	9 (24)
Female children >5 y old	9 (13)	3 (13)	5 (10)	5 (13)
Male children >5 y old	6 (9)	0 (0)	5 (10)	6 (16)
Other children ≤5 y old	7 (11)	2 (9)	6 (13)	2 (5)
Transmission in unvaccinated household, No. (%)^e^				
Overall	81	28	73	58
Women	23 (29)	9 (32)	22 (30)	18 (31)
Men	14 (17)	4 (14)	14 (19)	11 (19)
Female children >5 y old	4 (5)	1 (4)	4 (6)	2 (3)
Male children >5 y old	10 (12)	3 (11)	8 (11)	9 (16)
Children ≤5 y old	30 (37)	11 (39)	25 (34)	18 (31)
Transmission to children ≤5 y old in unvaccinated households, No. (%)				
Overall	30	11	25	18
No OPV exposure	13 (14)^c^	5 (5)	12 (13)^c^	8 (9)
≥1 OPV dose	17 (7)^c^	6 (2)	13 (5)^c^	10 (4)
3 IPV doses	9 (9)	5 (5)	8 (8)	7 (7)
4 IPV doses	16 (7)	5 (2)	13 (6)	9 (4)

Abbreviations: IPV, inactivated polio vaccine; OPV, oral polio vaccine; SD, standard deviation.

^a^For shedding duration among vaccines, we compared (1) OPV exposure versus no OPV exposure and (2) 3 versus 4 IPV doses.

^b^ *P* < .01.

^c^ *P* < .05.

^d^ *P* < .001.

^e^Percentage of the total number with shedding in unvaccinated households.

### Overall Community Transmission

Of the 1124 community contacts, only 7% shed OPV at ≥1 points (Figure 1). Transmission to unvaccinated households mirrored the amount of vaccination coverage in the area, with the 70% area experiencing the highest rate transmission and the 10% area experiencing the lowest.

Community contacts ≤5 years of age accounted for 30%–40% of transmissions, depending on OPV serotype (Table 2). Women were the next most likely to shed transmitted OPV, accounting for about 30% of transmissions across OPV serotypes (Table 2). Finally, men and children ≥5 years of age contributed similarly low amounts, each accounting for 14%–20% of the transmissions, depending on serotype (Table 2).

Transmissions were detected in similar proportions within each family member group. In unvaccinated households, 8% of all children ≤5 years old (30 of 370), 8% of all men (14 of 168), 7% of children >5 years old (14 of 206), and 6% of women (23 of 380) shed transmitted OPV (Table 2).

Prior OPV exposure seems to affect shedding for children ≤5 years old in unvaccinated households. Significantly fewer children with prior OPV exposure than without prior exposure shed OPV, 14% versus 7% (*P* = .04), respectively. The directionality of this difference is seen in all OPV serotypes but was significant only for serotype 2 (*P* = .01) (Table 2). Although the number of IPV doses did not seem to significantly affect transmission, the children who received 4 doses of IPV did show a nonsignificant decrease in shedding compared with those who received 3 doses (Table 2).

There were no significant differences in overall transmission between household contacts and unvaccinated participants (OR, 0.67; 95% CI, .37–1.20). Although household contacts in the 70% and 30% communities experienced more transmissions than those in the 10% community, transmission rates for OPV were comparable within the 70% and 30% communities. Within unvaccinated households, subjects in the 70% community were more than twice as likely to experience transmission as those in the 30% community (OR, 2.03; 95% CI, 1.05–3.93), and those in the 30% community had significantly higher chances of experiencing transmission than those in the 10% community (2.33; 1.14–4.74). These differences were not consistent across serotype. Within serotype, transmission of serotype 2 was most prevalent, with serotype 1 the least transmitted. Age, presence of a running toilet within the household, and household density were not significant in any of the models (Figure 1).

### Household Shedding and Transmission Over Time

**Figure 2.**
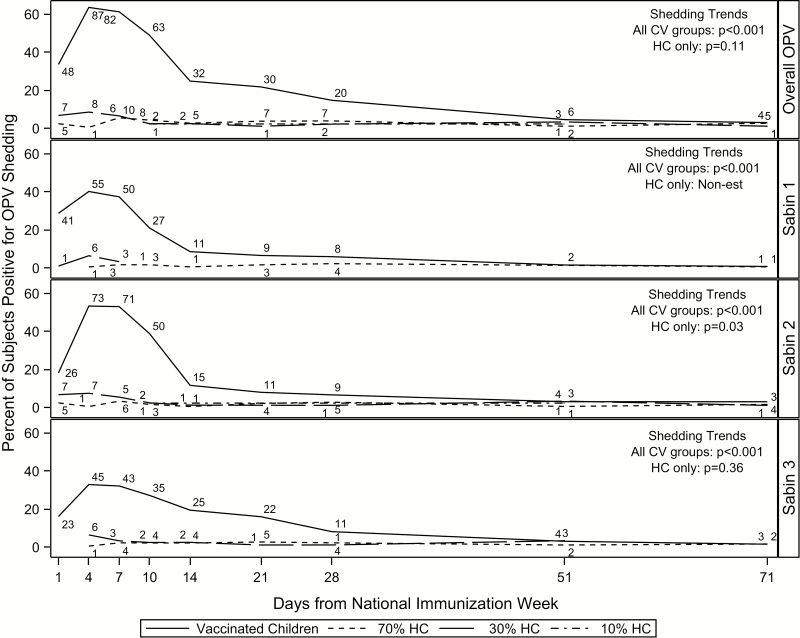
Shedding and transmission over time by CV group and serotype in vaccinated households. Day 0 is the day of OPV administration. Numbers above each line represent the number of subjects shedding at that time point. Abbreviations: CV, coverage area/vaccination group; HCs, household contacts; OPV, oral polio vaccine.

Not only did vaccinated children have the highest rates of shedding, but the duration of time between initial shedding and peak shedding was shorter, with peak shedding occurring earlier than the household contacts in the 70% coverage area (Figure 2 and Table 2). A more detailed depiction of household contact transmission over time is included in the Supplementary Material (Supplementary Figure 2). Peak transmission occurred on day 7 in the 70% coverage area and day 4 in the 30% area; because only 2 subjects in the 10% area experienced transmission, and on different days, there was not a peak day of transmission (Table 2). Peak transmission for serotypes 1 and 3 occurred much later among household contacts in the 70% coverage area, on days 28 and 21, respectively. Rates of shedding and transmission begin to stabilize around day 14. Similar to the overall results, shedding and transmission were most prevalent within Sabin 2.

Shedding duration was calculated using the first 28 days of the study for vaccinated children, using the first and last consecutive positive samples to avoid calculating artificially long shedding durations. Overall, vaccinated children shed OPV for a mean of 9.3 days (SD, 7.1 days) (Table 2). Whereas shedding durations were comparable for Sabin serotypes 1 and 2, Sabin serotype 3 had a longer shedding duration; overall, vaccinated children shed Sabin 1, 2, and 3 for a mean (SD) of 7.1 (5.3), 7.3 (5.4), and 11.02 (7.4) days (Table 2), respectively. Furthermore, vaccine history seems to be significantly associated with shedding duration for OPV-vaccinated children. Vaccinees with prior OPV exposure shed for significantly fewer days than those without prior OPV exposure, for a mean (SD) of 7.6 (5.7) versus 14.3 (8.4) days, respectively (*P* < .001). Similarly, vaccinees with 4 doses of IPV shed for significantly fewer days than those with 3 doses, for a mean (SD) of 6.9 (4.8) and 13.6 (8.3) days, respectively. These differences persist across all 3 Sabin serotypes (Table 2). Average duration of shedding by household contacts could not be calculated, because only 10 contacts shed at multiple, consecutive time points.

As expected, household contacts shed significantly less than vaccinated children over time (OR, 0.07; 95% CI, .03–.16) (Table 3). The transmission rate within the 70% coverage area was almost 8 times that of in 10% area (OR, 7.97; 95% CI, 1.44–44.10). Similarly, the rate within the 30% area was >10 times that of the 10% area (OR, 10.07; 95% CI, 1.78–57.05). Transmission rates between the 70% and 30% areas were comparable (overall OR, 0.79; 95% CI, .47–1.34). Shedding trends over time differed significantly across all groups (*P* < .001), but not within household contacts (*P* = .11) for overall OPV shedding and transmission. However, transmission trends over time within serotype 2 were significantly different (*P* = .03), suggesting that transmission over time varied across coverage areas (Figure 2). Results from the sensitivity analysis were comparable to the primary findings when samples were restricted to the first 28 days of the study (Supplementary Table 1).

**Table 3.** Pairwise Comparisons of Shedding/Transmission Over Time by Serotype

CV Group	Odds Ratio (95% CI)^a^			
	Overall OPV	Sabin 1	Sabin 2	Sabin 3^b^
Household contacts vs vaccinated children	0.07 (.03–.16)	0.07 (.02–.24)	0.09 (.04–.21)	0.21 (.11–.41)
Household contacts by coverage area				
70% vs 30%	0.79 (.47–1.34)	0.61 (.20–1.85)	0.59 (.31–1.12)	0.68 (.34–1.34)
70% vs 10%	7.97 (1.44–44.10)	5.93 (.83–42.16)	4.56 (.80–25.88)	Nonestimable
30% vs 10%	10.07 (1.78–57.05)	9.65 (1.25–74.24)	7.71 (1.34–44.26)	Nonestimable
Unvaccinated contacts vs household contacts	0.61 (.35–1.07)	0.67 (.27–1.64)	0.73 (.41–1.30)	0.47 (.30–.73)
Unvaccinated contacts by coverage area				
70% vs 30%	2.70 (1.51–4.85)	4.83 (1.37–17.08)	2.40 (1.31–4.39)	2.22 (1.14–4.30)
70% vs 10%	4.96 (2.76–8.93)	3.89 (1.15–13.09)	5.77 (3.06–10.90)	3.38 (1.77–6.46)
30% vs 10%	1.84 (1.01–3.35)	0.80 (.21–3.11)	2.41 (1.27–4.55)	1.53 (.80–2.92)

Abbreviations: CI, confidence interval; CV, coverage area/vaccination group; IPV, inactivated polio vaccine.

^a^Controlling for time, quadratic time, age, household density, and running toilets with a cluster effect for subject nested in household.

^b^Owing to zero transmission in the 10% area, pairwise comparisons within coverage were nonestimable.

### Community Transmission Over Time

**Figure 3.**
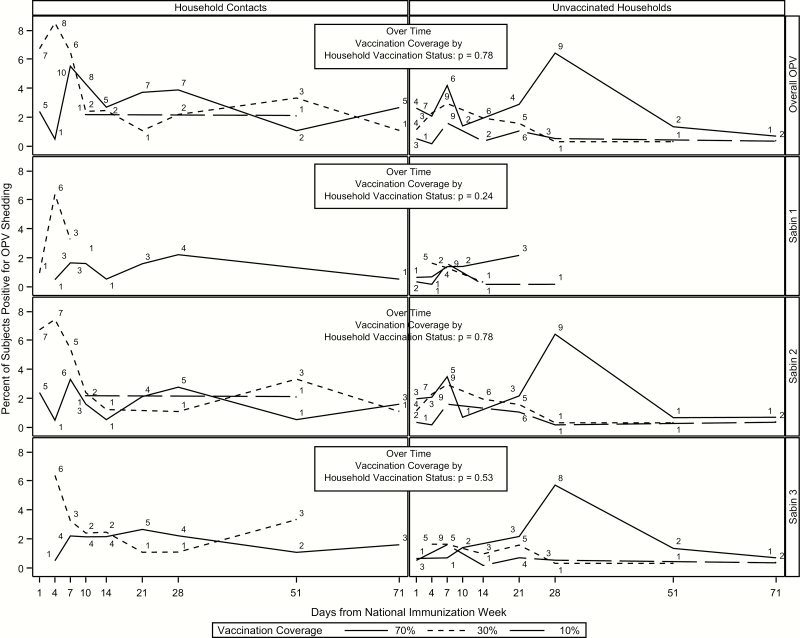
Transmission over time by group and serotype for household and community contacts. Numbers above each line represent the number of subjects shedding at that time point. Abbreviation: OPV, oral polio vaccine.

Transmission peaked latest in the unvaccinated subjects in the 70% community (7% of subjects at day 28) (Figure 3 and Table 2). Peak transmission occurred earlier in household contacts than in unvaccinated subjects, with the largest delay occurring in the 70% community (day 7 for household contacts and day 28 for unvaccinated subjects). Similar to the overall results, transmission was most prevalent within serotype 2 and least prevalent within serotype 1.

There were no significant differences in transmission between household contacts and unvaccinated subjects (OR, 0.61; 95% CI, .35–1.07) or over time by OPV coverage (Figure 3 and Table 3). Furthermore, there were no differences in transmission in household contacts between the 3 communities. However, among unvaccinated subjects, there were significant differences in transmission between the 70% and 30% (OR, 2.70; 95% CI, 1.51–4.85), 70% and 10% (4.96; 2.76–8.93), and 30% and 10% (1.84; 1.01–3.35) communities. All differences in transmission persisted across serotype except for the comparisons for the 30% versus 10% communities within serotypes 1 and 3.

### Time to First Shedding and Transmission Within Households

As expected, vaccinated children initially shed earlier than their household contacts. The median time to first shedding was 4 (range, 1–21) days for vaccinated children, 14 (1–75) days for the household contacts in the 70% coverage area, 7 (2–73) days for those in the 30% area, and 30.5 (10–51) days for those in the 10% area (Table 2). This large difference in median time for the household contacts in the 10% coverage area is probably due to the low levels of transmission in those households.

**Figure 4.**
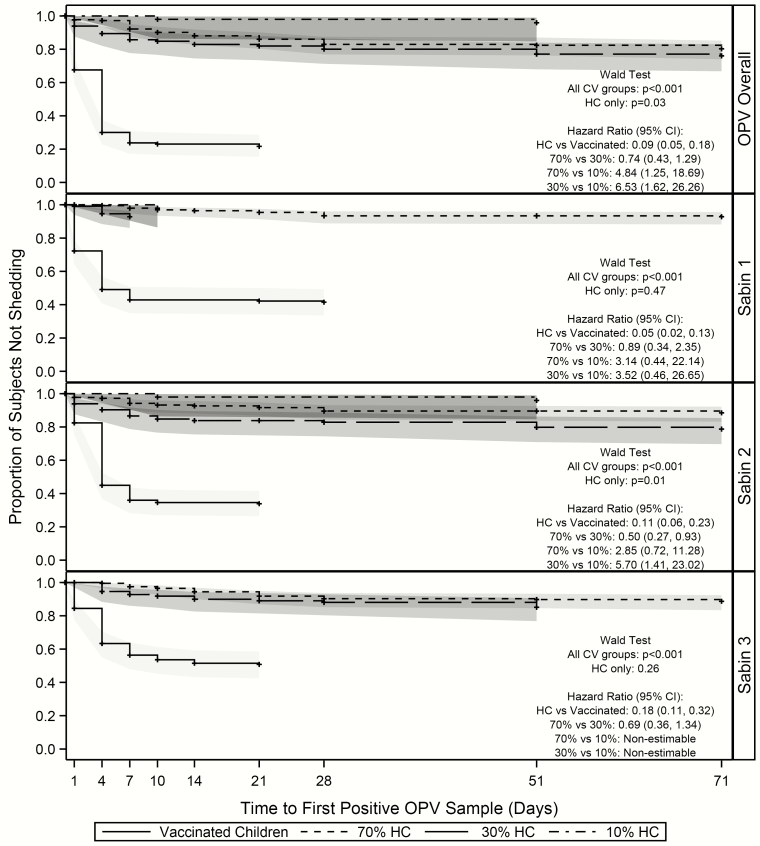
Time to-first shedding/transmission within vaccinated households. Abbreviations: CI, confidence interval; CV, coverage area/vaccination group; HCs, household contact; OPV, oral polio vaccine.

There were significant differences in time to first shedding and transmission of OPV overall across all CV groups (*P* < .001) and within the household contacts (*P* = .03). Similar findings were observed within the Sabin 2 serotype. Household contacts had a significantly lower risk than the vaccinated children of having their first positive sample early in the study (HR, 0.09; 95% CI, .05–.18). Household contacts in the 70% and 30% coverage areas were more likely to experience transmission than those in the 10% area (HR, 4.84 [95% CI, 1.25–18.69] and 6.53 [1.62–26.26], respectively). There was no significant difference in time to first transmission between the household contacts in the 70% and 30% areas (HR, 0.74; 95% CI, .43–1.29). Within serotypes, the hazard of experiencing first transmission was significantly different only between the household contacts in the 30% and 10% areas for Sabin serotype 2 (HR, 5.70; 95% CI, 1.41–23.02) (Figure 4).

### Time to First Community Transmission

The median time to first transmission for household contacts in the 70% community occurred 14 days after the NHW, approximately 1 week earlier than for their unvaccinated household counterparts (Table 2). The median time to first transmission was equal for household and community contacts in the 30% area, and >15 days later for household contacts in the 10% community (30.5 vs 14 days, respectively).

**Figure 5.**
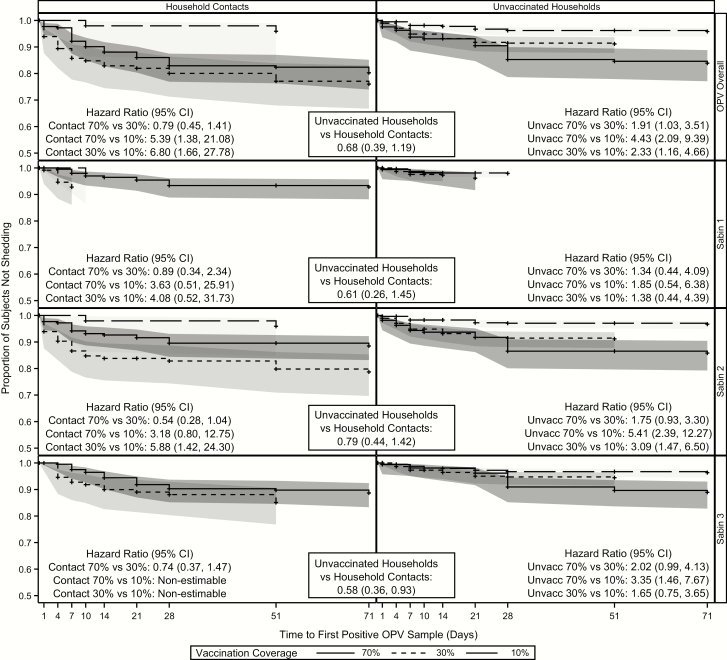
Time to first shedding/transmission for household and community contacts. Abbreviations: CI, confidence interval; OPV, oral polio vaccine.

There were no significant differences in the overall time to first transmission between unvaccinated households and household contacts (HR, 0.69; 95% CI, .39–1.19) (Figure 5). However, within unvaccinated households, all comparisons showed statistically significant differences; subjects in the 70% community experienced their first transmission almost twice as quickly as those in the 30% community (HR, 1.91; 95% CI, 1.03–3.51) and >4 times as quickly as those the 10% community (4.43; 2.09–9.39). Unvaccinated household contacts in the 30% community experienced their first transmission more than twice as quickly as those in the 10% community (HR, 2.33; 9% CI, 1.16–4.66). Within household contacts, subjects in both the 70% and 30% communities experienced their first transmission earlier than those in the 10% community (HR for 70% community, 5.39 [95% CI, 1.39–21.08]; HR for 30% community, 6.80 [1.66–27.78]). There were no differences in time to first transmission between household contacts of the 70% and 30% communities (HR, 0.79; 95% CI, .45–1.41). As with the previous analyses, transmission was most prevalent within serotype 2 and was similar to the overall transmission results.

## DISCUSSION

Our study demonstrated no differences in rates of intra- and interhousehold transmission of OPV in a community with primarily IPV-vaccinated children. Given the proximity of household contacts to vaccinated children, we expected household contacts to be at higher risk than community contacts for acquiring transmitted OPV. However, we did not find significant differences between household and community contacts, except for OPV serotype 3 transmission. This finding could also be a result of short shedding times, because the OPV-vaccinated children in our study were shown to shed for only 7–11 days, depending on OPV serotype.

Overall, 18% of all household contacts (67 of 380) had ≥1 positive sample at any time in the study. As expected, OPV was transmitted primarily to women in vaccinated households, who account for 51% of the overall intrahousehold transmission. Of all women, 21% shed OPV (34 of 162). This estimate is higher than a recent analysis of HIV-infected mothers in Zimbabwe, which found household transmission to only 5% of mothers with OPV-vaccinated children [20]. Other children in the household of a vaccinated child were also at high risk of shedding and accounted for 33% of the overall intrahousehold transmission. These results indicate that the most likely family members to shed OPV are primary caretakers or playmates of OPV-vaccinated children.

Unvaccinated households did demonstrate differences in time to acquisition of OPV relative to household contacts of vaccinees. Whereas community contacts began transmitting within a day after vaccination of the index child, as shown in Figures 3 and 4, and low levels of community transmission occurred rapidly, peak OPV transmission in community contacts was later than that of household contacts, both overall and for OPV serotypes 2 and 3 individually (Table 2). Most community transmissions occurred in children ≤5 years old and in women, similar to findings of previous studies demonstrating that children are at particularly high risk of OPV transmission [5, 21–23]. In future eradication and outbreak control efforts, children and possibly women should be targeted by interventions to interrupt household and community transmission, such as handwashing interventions to prevent fecal-oral transmission of OPV [24].

As expected, community OPV transmission was significantly associated with OPV coverage rates within each community. The 70% coverage area had higher overall community transmission than both the 30% and 10% areas, and the 30% coverage area also showed higher transmission rates than the 10% area, indicating transmission increases as the amount of OPV coverage increases. These results are mirrored in the transmission of serotype 2, where both the 70% and 30% communities showed higher transmission rates than the 10% community, and in the transmission of serotype 3, because the 70% community showed greater transmission than both the 30% and 10% communities. Serotype 1 showed no statistically significant differences by OPV coverage, as shown in Table 3. There was significantly higher risk of transmission over time in the 70% community than in the 30% and 10% communities, both overall and across all 3 serotypes, as well as higher transmission over time in the 30% community than in the 10% community, both overall and for OPV 2. These results indicate that transmission over time increases as OPV coverage increases in a community.

Our overall household transmission rates fell within transmission rates calculated from older studies, which found transmission rates ranging from 9% to 53% [5, 22, 25]. However, those proportions were calculated with fewer samples, with the largest group having only 76 participants, and with populations that included children with no prior polio immunization. Of the 3 OPV serotypes, serotype 2 was the most transmitted in both intra- and interhousehold transmission; 12.6% of household contacts and 6.5% of community contacts shed serotype 2, whereas serotypes 1 and 3 were shed by only 6.1% and 10.0% of household contacts and 2.5% and 5.2% of community contacts, respectively. The high prevalence of detected circulating VDPV serotype 2 may be linked to this increased transmissibility of serotype 2 [26]. These results are consistent with a prior study of OPV transmission from our group, which found serotype 2 to be the most transmitted OPV serotype in community circulation [6]. With the transition to bivalent OPV in 2016, transmissibility patterns of serotypes 1 and 3 may be disrupted in the absence of serotype 2. Additionally, the removal of OPV serotype 2 will result in shifting population immunity. This will make poliovirus surveillance critical in the prevention of future circulating VDPVs, particularly for serotype 2. This is best illustrated in a recent OPV transmission study in Bangladesh, which modeled the reintroduction of OPV serotype 2 in post-cessation birth cohorts, and which estimates that transmission in these cohorts could approach levels seen before widespread polio vaccination [27].

Literature on OPV transmission from the 1950s suggests that only a few hours of contact are required for OPV transmission [22, 25]. However, despite the apparent ease of transmission, only 7% of our community contacts shed transmitted OPV. These low numbers could be due to the short shedding duration of vaccinees and their contacts. Shedding duration in the current study was shorter than previously reported for OPV-vaccinated children. A previous study found OPV shedding up to 92 days after vaccination, while these children only shed for a little over a week [23]. Both IPV and OPV vaccination seemed to significantly affect OPV shedding duration. Children with prior OPV exposure shed for half as long as those with no prior exposure (7.6 vs 14.3 days, respectively) and children with 4 doses of IPV also shed half as long as those with 3 doses (6.9 vs 13.6 days). However, the decreased shedding could also be the result of age, because better vaccinated children are likely to be older, or some combination of IPV and OPV that reduced OPV shedding, because prior research has shown that a mixed schedule of IPV and OPV could affect mucosal intestinal immunity [28].

Only 10 household contacts and 3 community contacts shed transmitted OPV at multiple consecutive time points. As a result, average shedding duration for either group could not be accurately calculated. However, taking into account peak community transmission and the sample collection schedule, it is highly unlikely that shedding duration was >7 days. Furthermore, these short shedding times probably prevented prolonged transmission, as by day 71 only 1 or 2 community contacts were still OPV positive. This is particularly reassuring for the children ≤5 years old with transmission in these communities, who are all primarily IPV-vaccinated, because IPV has been shown to confer inferior mucosal immunity, compared with OPV [28, 29]. However, this short duration of shedding suggests reasonable intestinal immunity in the population. The children in this study shed for a shorter duration than primarily OPV-vaccinated Zimbabwean children in a prior analysis from our group, who were found to be shedding up to 92 days after OPV vaccination [30].

We expected there would be no differences in OPV transmission to household contacts when comparing the 3 villages. However, our models found differences both in overall transmission and in transmission of serotype 2. This is probably due to the increased presence of interhousehold transmission in the communities with high vaccination levels. In the overall model, both Capoluca (70% household OPV coverage) and Campo Grande (30%) had significantly higher transmission to household contacts than Tuxpanguillo (10%). The overall serotype 2 model also found higher transmission of serotype 2 in the 30% community compared with the 70% and 10% communities.

Although differences in our over time models were not significant overall, there differences were detected in the transmission of Sabin serotype 2 over time. The differences in transmission detected when comparing the 10% community could be attributable to the low transmission rate in that community, because only 2 intrahousehold contacts had positive samples. However, the differences in overall Sabin 2 transmission between the 70% and 30% communities are unusual. In a prior 1950s study, improved socioeconomic status was found to significantly decrease transmission to household contacts [22]. As mentioned above, the social development wealth index showed that the 70% community had the highest level of wealth, compared with the 30% and 10% communities. The differences in our intrahousehold transmission models between the 30% and 70% communities could be attributed to this socioeconomic difference.

Our study has some key limitations. First, we assume that transmission to household contacts is the result of secondary transmission from the household’s OPV-vaccinated child, because we are certain that the original sources of OPV transmission in these households are the vaccinated children. However, without contact information or viral genome sequencing, we cannot confirm whether these contacts were infected via inter- or intrahousehold transmission or which contacts propagated transmission. Second, samples were not collected daily, so transmission and shedding could have been missed in the gaps between sample collections, resulting in underestimated transmission. Third, men were more likely than women to refuse to participate, usually owing to the number of samples requested for the study. Thus, 59% of our community contacts and 64.7% of household contacts were women, and transmission to men was likely underestimated.

Our study also has multiple strengths. First, our results here include samples from 148 vaccinated children, 380 household contacts, and 1124 unvaccinated community contacts, giving us a larger sample size than many older studies looking at OPV transmission. Second, the only children vaccinated in these 3 communities during the February 2015 NHW were the 155 vaccinated children in our study. No other OPV was introduced into these communities until the May 2015 NHW, so the transmission seen in our study can be directly linked to our vaccinees. Third, because children were required to have ≥2 doses of IPV to be vaccinated, this mimics possible future environments of OPV transmission, with global transition to primary use of IPV. Finally, this study is unique, as few OPV transmission studies have been conducted, and we were only able to find one similar manuscript that was recently published [27].

In conclusion, we found that OPV coverage was significantly associated with interhousehold transmission, and that household and community transmission rates were similar. Women and children other than the vaccinated child were most likely to shed transmitted OPV, indicating that primary caretakers and playmates of vaccinated children are at highest risk of OPV transmission. Interrupting transmission to these contacts, through handwashing interventions, for example, could be critical in containing future outbreaks. Future analyses should assess contact information and genetic sequencing to carefully track infections throughout communities and clarify the propagation dynamics of transmission within households.

Low levels of transmission to community contacts occurred within a day of vaccination, even in the 10% community, confirming that transmission occurs quickly after OPV administration. Whereas most participants were shown to shed transmitted OPV for less than a week, this short time was sufficient to propagate community transmission as long as 71 days after vaccination. Finally, although all serotypes were shed throughout the study, serotype 2 was seen in the most participants, indicating that it is the most transmissible of all 3 OPV strains. Because WPV serotype 2 has been eradicated, our data support the transition from trivalent to bivalent OPV that occurred in 2016. Further understanding of poliovirus transmission dynamics would aid in postcertification efforts, when IPV-only immunization regimens are expected.

## Supplementary Data

Supplementary materials are available at *Clinical Infectious Diseases* online. Consisting of data provided by the authors to benefit the reader, the posted materials are not copyedited and are the sole responsibility of the authors, so questions or comments should be addressed to the corresponding author.

## Supplementary Material

Supplementary_Figure_1

Supplementary_Figure_2

Supplementary_Tables

## Notes

***Acknowledgments.*** We thank the families who participated in this study, the large field teams in Mexico that performed data and sample collection, and the broader team at Stanford University, including Aisha Talib and Jennifer Lee Kang.

***Financial support.*** This study was supported by the Bill & Melinda Gates Foundation (grant OPP1116910).

***Supplement sponsorship.*** This article appears as part of the supplement “Polio endgame and beyond: Vaccine choices, transmission dynamics, and surveillance implications,” sponsored by The Bill & Melinda Gates Foundation.

***Potential conflicts of interest.*** L. G. G., L. F. R., R M. C., E. F. G., G. D. S., and S. C. Q. reports grants and personal fees from the Bill & Melinda Gates Foundation and Stanford University during the conduct of the study, and grants and nonfinancial support from GlaxoSmithKline Biologicals, outside the submitted work. L. P. C. H. reports grants from GlaxoSmithKline, outside the submitted work. Y. A. M. reports grants from the Bill & Melinda Gates Foundation, during the conduct of the study, and personal fees and other support from Pfizer, outside the submitted work.
