# Supplementary figures and images for: Characterization of Household and Community Shedding and Transmission of Oral Polio Vaccine in Mexican Communities With Varying Vaccination Coverage
Source: Clin Infect Dis. 2018 Oct 30;67(Suppl 1):S4–S17. doi: 10.1093/cid/ciy650 (PMC6206120; doi:10.1093/cid/ciy650)

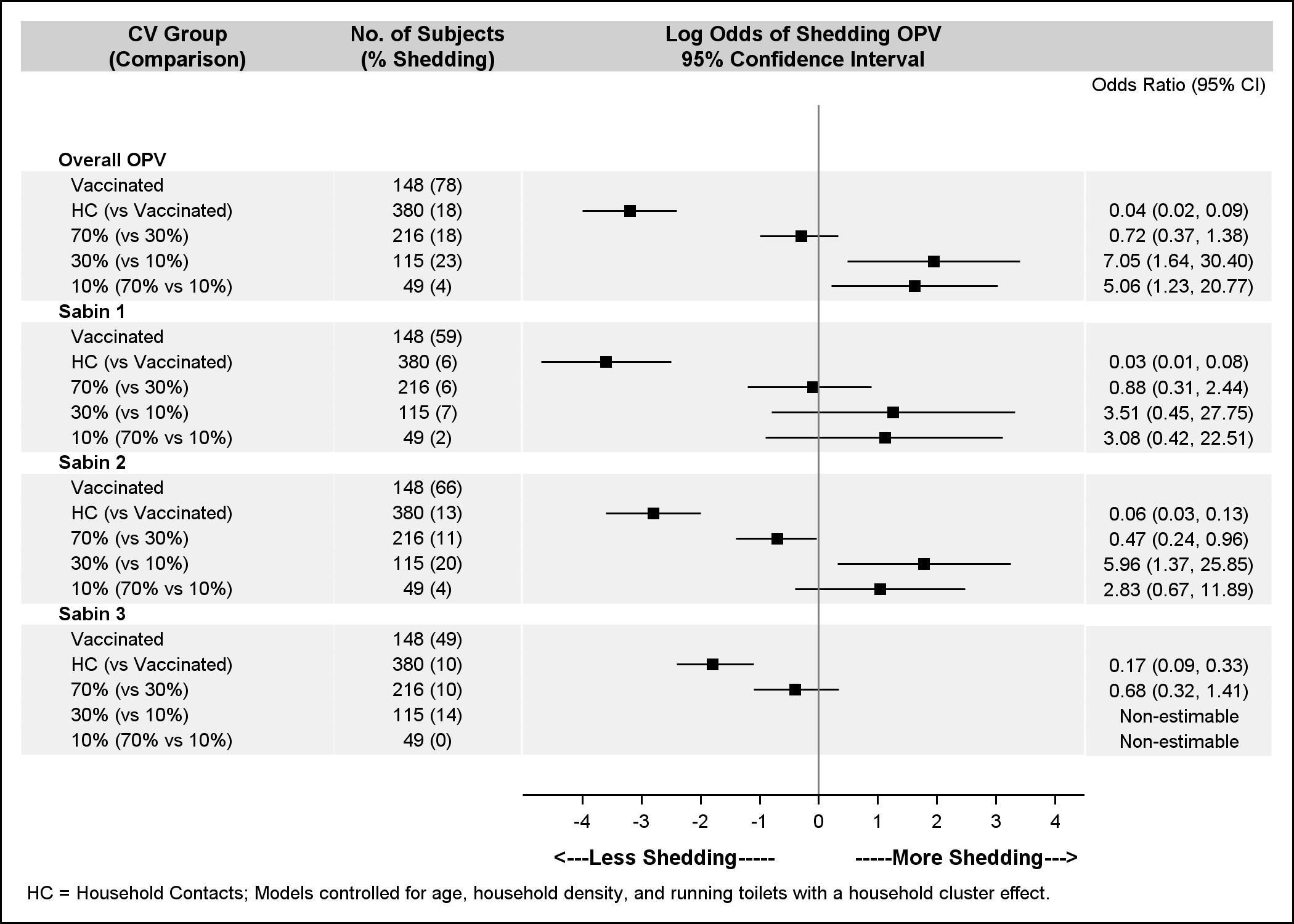

Supplement: Supplementary_Figure_1 [file ciy650_suppl_supplementary_figure_1.jpeg]

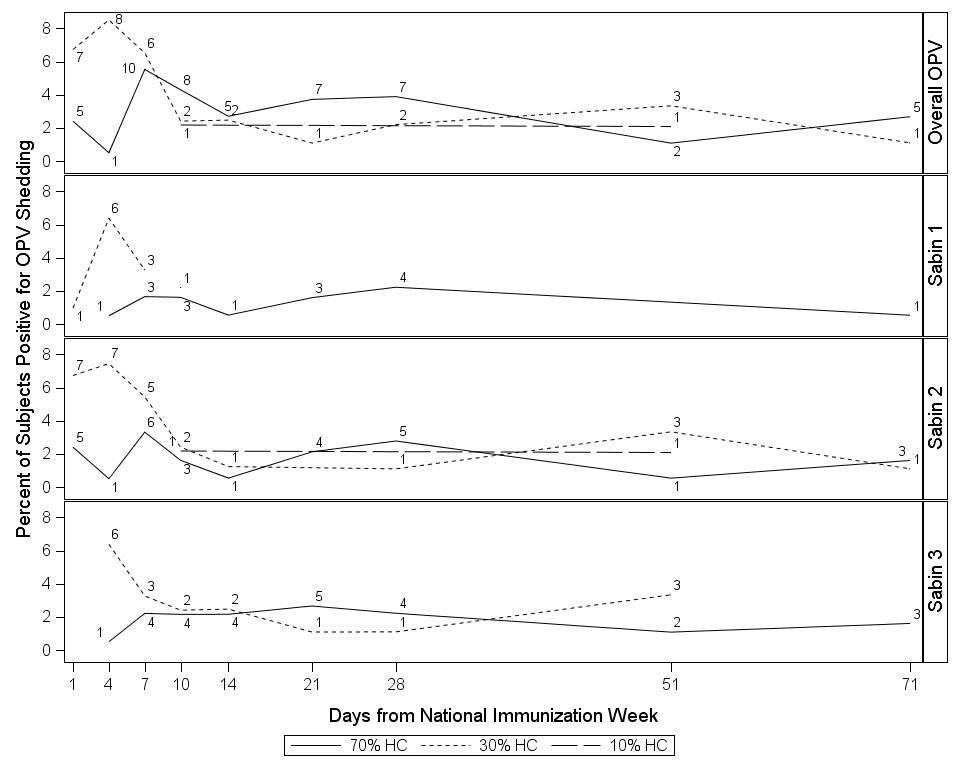

Supplement: Supplementary_Figure_2 [file ciy650_suppl_supplementary_figure_2.png]
