# Supplementary material for: Characterization of Household and Community Shedding and Transmission of Oral Polio Vaccine in Mexican Communities With Varying Vaccination Coverage
Source: Clin Infect Dis. 2018 Oct 30;67(Suppl 1):S4–S17. doi: 10.1093/cid/ciy650 (PMC6206120; doi:10.1093/cid/ciy650)
Supplement: Supplementary_Tables [file ciy650_suppl_supplementary_tables.docx]

**Supplementary Table 1.** Pairwise Comparisons of Shedding / Transmission Over First 28 Days by Serotype

|  | Odds Ratio  (95% Confidence Interval)^†^ | | | |
| --- | --- | --- | --- | --- |
|  | Overall OPV | Sabin 1 | Sabin 2 | Sabin 3^‡^ |
| CV Group |  |  |  |  |
| Household Contacts (HC) vs Vaccinated Children | 0.04  (0.02, 0.09) | 0.05  (0.01, 0.17) | 0.06  (0.02, 0.15) | 0.13  (0.07, 0.23) |
| 70% HC vs 30% HC | 0.77  (0.42, 1.42) | 0.69  (0.28, 1.72) | 0.75  (0.27, 2.12) | 0.66  (0.31, 1.42) |
| 70% HC vs 10% HC | 13.54  (1.89, 97.22) | 4.04  (0.45, 36.12) | 7.81  (1.07, 57.33) | Non-estimable |
| 30% HC vs 10% HC | 17.53  (2.36, 130.17) | 5.85  (0.62, 55.27) | 10.36  (1.20, 89.22) | Non-estimable |

^†^ Controlling for time^2^, age, household density, and running toilets with a cluster effect for subject nested in household.

^‡^ Due to zero transmission in the 10% area, pairwise effects were non-estimable.
